# Plasma and Urinary TMAO and Methylamine Responses to Meat and Egg Ingestion: Links to Gut Microbiota Composition in Subjects With and Without Metabolic Syndrome

**DOI:** 10.3390/nu17233719

**Published:** 2025-11-27

**Authors:** Mohammed E. Hefni, Anders Esberg, Patrik Hellström, Ingegerd Johansson, Cornelia M. Witthöft

**Affiliations:** 1 Department of Chemistry and Biomedical Sciences, Linnaeus University, 392 31 Kalmar, Sweden; cornelia.witthoft@lnu.se; 2 Food Industries Department, Faculty of Agriculture, Mansoura University, P.O. Box 46, Mansoura 35516, Egypt; 3 Department of Odontology, Umeå University, 901 87 Umea, Sweden; anders.esberg@umu.se (A.E.); ingegerd.johansson@umu.se (I.J.); 4 Department of Health and Caring Sciences, Linnaeus University, 392 31 Kalmar, Sweden; patrik.hellstrom@lnu.se

**Keywords:** egg, meat, TMAO, gut microbiota, metabolic syndrome

## Abstract

Background/Objectives: Trimethylamine *N*-oxide (TMAO), a gut microbiota-derived metabolite from L-carnitine and choline (abundant in meat and eggs), is linked to CVD and T2D. This study investigated whether TMAO responses to animal-based foods differ between individuals with and without metabolic syndrome (MetS), in relation to their gut microbiota composition. Subjects/Methods: In a randomized crossover trial, 12 MetS (≥3 criteria according to the Adult Treatment Panel III: elevated waist circumference, fasting glucose, triglycerides, and blood pressure or reduced HDL cholesterol) and 21 non-MetS subjects consumed two test meals (3 hard-boiled eggs or 170 g meat balls) after overnight fasting, with ≥1-week washout. Blood was collected at baseline and 0.5, 1, 2, 4, and 6 h postprandially; urine was collected over 6 h. Fecal samples (collected pre-first day of intervention) underwent 16S rRNA sequencing. Plasma and urinary TMAO, TMA, choline, and carnitine were quantified using UPLC-MS/MS. Results: MetS subjects exhibited a non-significant trend towards higher incremental AUCs for plasma TMA, TMAO, choline, and carnitine after consuming both foods, with a 30–50% higher urinary TMAO excretion (but similar for TMA) versus non-MetS subjects. This exploratory analysis also indicated that MetS subjects had reduced gut microbial diversity, featuring decreased *Blautia glucerasea* (butyrate producer) and increased *Ruminococcus torques* (pro-inflammatory), a profile associated with inflammation but not TMA production. Conclusion: No significant increase in plasma methylamines after choline and carnitine challenge was observed in subjects with MetS compared with non-MetS. In MetS subjects (without CVD and T2D), gut microbiota composition was characterized by increased pro-inflammatory bacteria rather than TMAO-generating bacteria. The lack of statistical significance with regard to plasma TMAO response could be due to an insufficient sample size rather than the absence of an effect. Nevertheless, the observed elevation might still be clinically relevant, supported by concurrent differences in microbiota composition. These preliminary findings warrant validation in larger cohorts due to sample size limitations.

## 1. Introduction

Metabolic syndrome (MetS) is a cluster of interrelated conditions such as obesity, hyperglycemia, dyslipidemia, hypertension, and hyperuricemia, which collectively increase the risk of type 2 diabetes (T2D), cardiovascular disease (CVD), stroke, and myocardial infarction [1]. According to the National Cholesterol Education Program Adult Treatment Panel III (NCEP ATP III), an individual is diagnosed with MetS when they meet at least three of the following criteria: elevated waist circumference, high fasting plasma glucose, elevated triglycerides, low levels of high-density lipoprotein cholesterol, or high blood pressure [2]. The clinical significance of MetS lies in its capacity to increase the risk of T2D, stroke, myocardial infarction, and CVD by 2- to 5-fold, independent of prior cardiovascular events [1]. While genetic variants and lifestyle factors, such as diet and physical inactivity, are well-established contributors to MetS pathogenesis, there is growing evidence underscoring the gut microbiota as a pivotal modulator of metabolic health [3]. The human microbiome is predominantly composed of *Firmicutes* (Gram-positive) and *Bacteroidetes* (Gram-negative), accounting for 60–80% and 20–30% of the microbiota, respectively, with smaller proportions of *Proteobacteria* and *Actinobacteria* [4]. A diverse microbiota composition has been identified as a protective factor against metabolic diseases, including obesity, MetS, and T2D [5]. In contrast, reduced microbial diversity, a hallmark of dysbiosis, has been strongly linked to obesity, insulin resistance, and chronic inflammation—key features of MetS [5]. Dysbiosis of the gut microbiota has been shown to be involved in the pathogenesis of MetS through several mechanisms, including increased intestinal permeability, chronic low-grade systemic inflammation, and alterations in dietary energy harvesting [6,7]. Disrupted production of short-chain fatty acids (SCFAs), key fermentation byproducts of gut microbial metabolism, is associated with the development of insulin resistance and obesity in MetS [6,7]. Furthermore, impaired bile acid metabolism exacerbates dyslipidemia and insulin resistance, highlighting the multifaceted role of the gut microbiota in MetS progression [6,7].

Trimethylamine *N*-oxide (TMAO), a gut microbiota-dependent metabolite derived from dietary choline, which is abundant in eggs, and L-carnitine, which is abundant in red meat, is of particular interest [8,9,10]. Elevated plasma TMAO levels have been associated with atherogenesis through mechanisms including impaired reverse cholesterol transport, promotion of foam cell formation, and induction of pro-inflammatory cytokines (e.g., TNF-α, IL-6, CRP) [8]. TMAO is generated via microbial conversion of choline and L-carnitine to trimethylamine (TMA), which is oxidized in the liver by flavin monooxygenases (FMOs) following absorption [8,9,10]. Microbial TMA generation from dietary sources is described through two established primary pathways. Firstly, choline-to-TMA conversion occurs by several taxa, e.g., *Anaerococcus hydrogenalis*, *Clostridium* species (*asparagiformis*, *hathewayi*, *sporogenes*), *Desulfovibrio desulfuricans*, *Escherichia fergusonii*, *Klebsiella pneumoniae*, and *Proteus penneri* by the glycyl radical enzyme choline TMA-lyase (CutC) and its activator (CutD) [11]. Secondly, carnitine to TMA conversion is mediated by *Acinetobacter baumannii*, *Burkholderia* spp., *Cupriavidus* spp., *Pseudomonas* spp., *Shigella* spp., *Sporosarcina* spp., *Stenotrophomonas* spp., *Yersinia* spp., and *Yokenell* spp. via the Rieske-type oxygenase/reductase CntA/B [12]. On the other hand, *Bacteroidetes* lack the capability of TMA production [13,14,15,16]. However, although the role of the gut microbiota in TMAO production is evident, the effect of the microflora composition in relation to TMAO production from dietary sources in early MetS, which is defined as MetS prior to progression to CVD and T2D [17,18] and reported to be associated with altered gut microbiota profile, is not well-investigated.

Therefore, the aim of this study was to investigate postprandial TMAO concentrations in the plasma and urine of subjects with and without MetS after ingestion of choline- and L-carnitine-rich foods (eggs and meat, respectively) in association to their gut microbiota profiles.

## 2. Materials and Methods

### 2.1. Subjects

A total of thirty-three subjects (aged 18–75 y) were recruited from the student/staff population of Linnaeus University (Kalmar, Sweden) and the surrounding community through advertisements on the university website and in a local newspaper between January–May 2020 and December 2022–May 2023 (interrupted by COVID-19). The eligibility criteria required subjects to be non-smokers; not pregnant, breastfeeding, or planning pregnancy; not taking antibiotics or probiotics two months before recruitment; not taking nutritional supplements two weeks before the study period; and not following a special diet (e.g., vegan, vegetarian, weight loss). Additionally, subjects could not participate in another study. The study recruited 12 individuals with early MetS (free of CVD (self-reported) and T2D) and 21 without MetS (non-MetS). A diagnosis of MetS was based on the NCEP-ATP III criteria, which requires ≥3 of the following: elevated waist circumference (M > 102 cm, F > 88 cm), fasting plasma glucose ≥ 5.6 mmol/L or medication, triglycerides > 1.7 mmol/L or medication, low HDL-cholesterol (M < 1.03 mmol/L, F < 1.29 mmol/L) or medication, or hypertension (≥130/85 mm Hg) or medication. Normal HbA1c within the age-related reference range was required to exclude diabetes. Non-MetS subjects should be free from self-reported disease symptoms and possess fasted plasma glucose, hemoglobin, liver status (aspartate transaminase, alanine transaminase, and γ-glutamyl transferase activity), blood status, lipid profile (P-triglycerides, P-HDL-cholesterol, and P-cholesterol), kidney function (P-creatinine value within age-related reference range) in the normal biochemical range, and a BMI between 18.6 and 29.0 kg/m^2^. The study was approved by the Swedish Ethical Review Authority (Dnr: 2019-04354). All study subjects signed an informed consent form after being informed about the study.

### 2.2. Study Design

Two animal-derived TMAO precursor test foods (three hard-boiled eggs or 170 g meatballs) were administered in a randomized crossover order on two test days, separated by at least a two-week washout period. Dietary intake and gut microbiota stability were not controlled or monitored during this period. Subjects were asked to maintain their normal dietary and exercise habits throughout the study. The day before the test session, subjects were asked to avoid consuming grapefruit juice and indole-containing vegetables (i.e., broccoli, Brussels sprouts, cabbage, cauliflower), as these foods can decrease FMO3 enzyme activity and alter TMAO metabolism [19]. Subjects were also asked to eat a similar evening meal on both occasions. A power calculation (G*Power 3.1.9.3, α < 0.05, 80% power, two-sided) estimated that 17 subjects per group would be sufficient to detect a 20% difference in plasma TMAO levels [19,20]. To account for potential dropouts, the number of subjects was set to 20 per group. However, despite extending the age limit from 65 to 75 years, only 12 MetS subjects were successfully recruited. For the inclusion screening, subjects were asked to fast overnight and to arrive at the study center between 07:00 and 08:00 a.m. for measurements of body weight, height, waist circumference, and blood pressure and blood sampling. Plasma was analyzed for glucose, liver enzymes, lipid profile, creatinine, and blood for HbA1c directly using standard methods at the Department of Clinical Chemistry and Transfusion Medicine, Diagnostic Center, County Hospital in Kalmar. Further, a complete blood count was conducted using a Swelab Alfa Plus Analyzer (Boule Diagnostics, Spånga, Sweden) at the biochemistry laboratory at Linnaeus University, Kalmar.

Subjects returned to the study center twice at 07:00–08:00 a.m. after overnight fasting and stayed until 13:00–14:00 for the test sessions. On each test day upon arrival, subjects were asked to collect a baseline spot urine sample. A baseline blood sample was collected following the insertion of a standard intravenous catheter. Body weight, length, waist circumference, and blood pressure were measured. Subjects were instructed to eat one of the randomly allocated test foods (3 eggs or 170 g meatballs) with free access to water within a 15 min period. A series of blood samples (3 mL in EDTA each) was collected at 30 min and 1, 2, 4, and 6 h post-dose. At 4.5 h, subjects were provided with a standardized snack (170 g applesauce), and they had free access to water throughout the day. Subjects were asked to collect post-dose urine over a 6 h period. Blood samples were immediately centrifuged at 2000× *g* for 10 min and plasma was transferred to 1.5 mL Eppendorf tubes and stored at −80 °C until analysis.

Subjects were provided with a specimen container (DNA/RNA shield-fecal collection tube (BioSite-R110, Nordicbiosite, Nordic BioSite AB, Täby, Sweden)) and a thermoisolated bag for transport and were asked to collect a stool sample on the day before the first intervention day. Subjects were instructed on how to collect the stool sample. Samples were stored at −20 °C until being transferred to −80 °C within a week from collection.

### 2.3. Analysis of TMAO and Related Metabolites in Clinical and Food Samples

The analysis of TMAO, TMA, betaine, choline, L-carnitine, acetyl-L-carnitine, and creatinine in clinical and food samples was carried out according to previously described method [21]. In brief, total choline was extracted from freeze-dried food using acidic hydrolysis, while betaine and carnitines were extracted with water by vortexing and centrifugation. Protein in plasma, urine, and food extracts (25 µL) was precipitated with methanol. Deuterated internal standards were added to samples before derivatization using iodoacetonitrile. The reaction was stopped by adding formic acid. Samples were centrifuged and aliquots of the supernatant were transferred to HPLC vials for analysis using an Agilent 1260 II UPLC system coupled to an Agilent G6495C triple-quadrupole MS equipped with AJS-ES ionization (Agilent Technologies, Santa Clara, CA, USA). Methylamines (1 µL injection volume) were separated on a neutral ACE UPLC-HILIC column using an isocratic mobile phase of 70% ammonium formate (10 mmol/L) and 30% acetonitrile at column temperature of 25 °C and a flow rate of 0.2 mL/min within a 6 min run time.

### 2.4. Microbiota Analysis

Microbiota analysis, including DNA extraction and full-length 16S rRNA gene sequencing, was described in detail by Hefni et al. 2025 [22]. In summary, fecal DNA was extracted from 250 mg stool using the DNeasy PowerSoil Pro Kit (QIAGEN, Kista, Sweden). The DNA quality was assessed using a NanoDrop 1000 spectrophotometer (Thermo Fisher Scientific, Uppsala, Sweden) and the quantity was estimated by the Qubit 4 Fluorometer (Invitrogen, Thermo Fisher Scientific, Waltham, MA, USA). A commercial DNA mock community (ZymoBIOMICS Microbial Community DNA Standard, D6305, Nordic Biosite, Stockholm, Sweden) was used as a positive control and ultrapure water as negative control. Full-length 16S rRNA (V1–V9) amplicons (~1465 bp) were generated, barcoded with the Native Barcoding Kit 96 V14, and sequenced on a GridION nanopore sequencer using a R10.4.1 flow cell (Oxford Nanopore Technologies, Oxford, UK). Reads were base-called and demultiplexed using MinKNOW/Dorado (Oxford Nanopore Technologies) and Porechop (version 0.2.4, https://github.com/rrwick/porechop, accessed on 1 February 2025), retaining high-quality reads (Q > 10, 1350–1800 bp). Taxonomic classification was performed with the Emu pipeline against the RDP v11.5 and NCBI taxonomy databases, followed by filtering low-abundance taxa and rarefying data to 28,848 reads. Diversity metrics, including species richness, Shannon index, and Bray–Curtis distances, were computed using the MicrobiotaProcess package in R.

### 2.5. Statistical Analysis

All statistical analyses were performed using R (RStudio 2024.04.2-764), and *p*-values < 0.05 were considered to indicate a statistically significant difference. Data was presented as the mean ± SD. Due to the non-normal distribution of the data, a nonparametric ANOVA-type test was applied using the nparLD::f1.ld.f1() function from the nparLD package, which accounts for repeated measures without assuming normality. Metabolite concentration was modeled as the response variable, with time as a within-subject factor and subject ID as a random effect. The statistical output included rank means for each time point and *p*-values from the ANOVA-type test, assessing whether metabolite levels significantly change over time. The Mann–Whitney U test (Wilcoxon Rank-Sum Test) was used to compare TMAO and related metabolite responses in post-dose urine between groups. To control the False Discovery Rate (FDR) across the multiple comparisons performed on the iAUC and time point data, the Benjamini–Hochberg procedure was applied to the resulting *p*-values. Corrected *p*-FDR < 0.05 were considered to indicate a statistically significant difference. For gut microbiota, Orthogonal Partial Least Squares Discriminant Analysis (OPLS) regression and Bray–Curtis dissimilarity index comparisons were run for microbiota comparisons and to identify the differing taxa between the two groups. The MaAsLin3 (Microbiome Multivariable Associations with Linear Models) [23] in R studio [24] was used to identify microbial taxa associated with having a MetS and non-MetS status in a multivariable generalized linear model regression which included age, sex, and total number of reads. Data transformations and nonparametric tests were employed when necessary to meet analysis assumptions.

## 3. Results

The baseline characteristics of subjects with and without MetS have been previously described [22]. Briefly, healthy subjects possessed demographics and laboratory tests values within the normal range, whereas, all values, excluding age, height, and P-cholesterol, were significantly higher in the MetS group [22]. The average choline content in meatballs and eggs was 38 ± 1.7 and 213 ± 4.7 mg/100 g fresh weight, respectively, while betaine content was 9.0 ± 0.1 and 1± 0.2 mg/100 g fresh weight, respectively (Table 1). L-Carnitine was present in meatballs (13 ± 0.5 mg/100 g, fresh weight) but was not detected in eggs (n = 6, three samples in duplicate analysis) (Table 1).

At screening, fasting plasma TMAO, TMA, choline, betaine, and acetyl-L-carnitine concentrations did not significantly differ between the MetS and non-MetS groups, whereas L-carnitine was significantly higher (*p* = 0.0191) in individuals with MetS.

**Figure 1:**
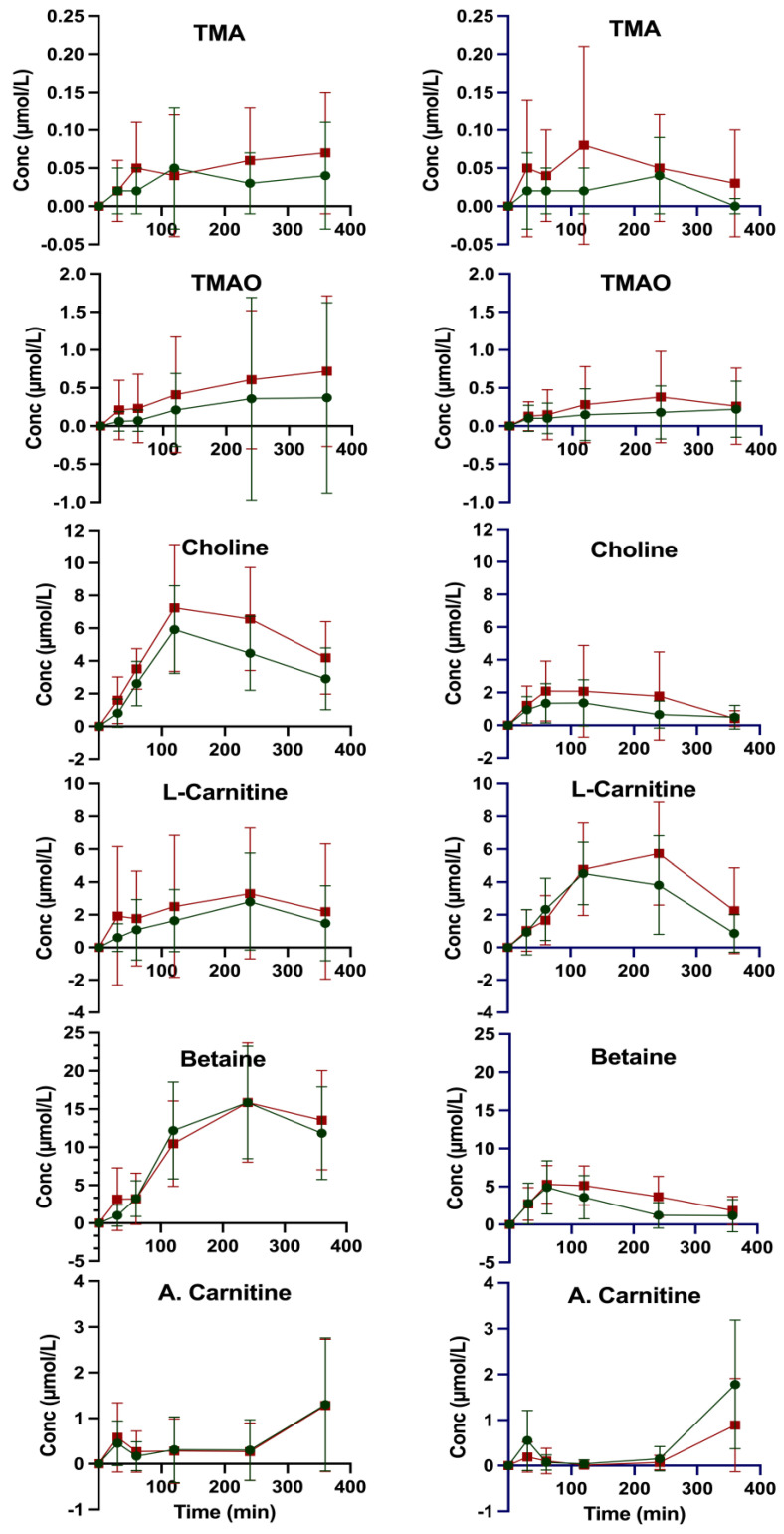
Incremental plasma AUC of methylamines in subjects without (green, n = 21) and with MetS (red, n = 12) after ingestion of 3 hard-boiled eggs (≈170 g) (**left side**) or 170 g meatballs (**right side**). A. Carnitine: Acetyl-L-carnitine.

After ingesting the test foods, either eggs or meatballs, individuals with MetS showed a non-significant trend for increased incremental area under the curve (iAUC) for TMA, TMAO, L-carnitine, and choline compared to non-MetS individuals (Figure 1). Plasma TMAO peaked at 4 h after meat ingestion in both groups, but after egg ingestion the maximum concentration was not reached until 6 h (Figure 1, Table 1). The shape of the TMA AUC varied depending on the type of ingested food as well as between groups (Figure 1).

The levels of TMAO excreted with post-dose urine over 6 h increased by 30–50% in subjects with MetS, whereas TMA excretion was similar across both groups (Table 2).

**Figure 2:**
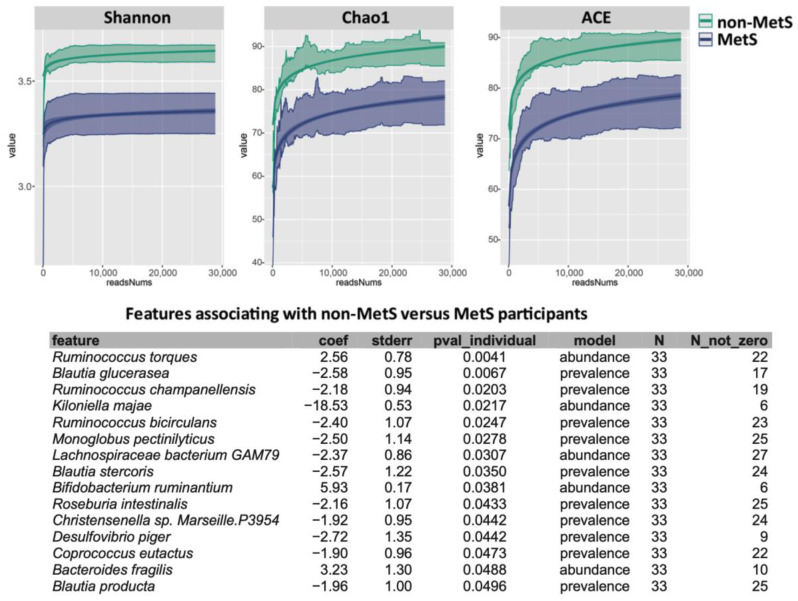
Gut microbiome Alpha diversity indices (Shannon, Chao1, and ACE) and associated features in MetS Status. Figure reproduced from our earlier publication (Figure S1 in Hefni et al. 2025 [22]).

Subjects without MetS had a higher diversity and richness of microbiota, as seen through the higher Shannon, Chao1, and ACE indices (Figure 2). The most pronounced microbial differences between groups were observed for *Ruminococcus torques*, which showed a 3.8-fold elevated level in MetS (*p* = 0.004), and the *Blautia glucerasea*, which showed an increased prevalence in the non-MetS group with an odds ratio (OR) (95% CI) of 15.1 (2.3–100.2) (*p* = 0.007).

## 4. Discussion

This study aimed to investigate postprandial TMAO and its related metabolites in plasma and urine following the ingestion of choline- and L-carnitine-rich foods (eggs and meatballs) in relation to gut microbiota profiles among subjects with and without MetS. MetS is a condition linked to altered gut microbiota composition. The inclusion of individuals with MetS—apparently free of T2D, CVD, or kidney dysfunction—minimizes the confounding effects of advanced comorbidities that can independently alter circulating methylamines [20,25,26].

Our findings revealed a trend of higher TMAO levels in the plasma and urine of subjects with MetS compared to the non-MetS group after equivalent dietary intakes of choline (eggs) and carnitine (meatballs). Although the differences in plasma TMAO concentration between both groups did not reach statistical significance—this outcome may be affected by the relatively small sample size particularly within the MetS group limiting the statistical power to capture significant differences—the observed trend in the MetS group may still be of interest, particularly when considering the differences in gut microbiota composition. These findings highlight the importance of interpreting the data within the context of sample-size limitations and emphasize the need for larger studies in MetS subjects (free of CVD and T2D).

Previous studies have shown an inconsistent link between egg consumption and plasma TMAO concentrations. While some studies have reported a significant increase in plasma TMAO levels following egg ingestion [15,27], others do not [19,28,29]. Miller et al. [27] observed an increase in plasma TMAO levels following egg consumption and further revealed that approximately 11–15% of dietary total choline from an egg-containing meal can be converted into TMAO. However, the extent to which egg consumption raises TMAO can be influenced by differences in study design, population characteristics, gut microbiota composition, and the metabolic capacity of the FMO3 enzyme. Eggs are rich in choline, primarily present as phosphatidylcholine which is not considered to be a suitable substrate for TMA-generating bacteria [28,30]. The conversion of phosphatidylcholine from eggs into TMA is a two-step process. It commences with the conversion to choline by the enzyme phospholipase D, followed by the conversion to TMA through the enzyme choline TMA lyase [9,16]. While the 6-hour sampling time in the current study was chosen based on results from previous studies suggesting that TMAO peaks within this period [15,19], we found that plasma TMA and TMAO levels had not peaked within six hours following egg ingestion (Figure 1). This is consistent with findings by Miller et al. [27], and underlines the necessity to extend sampling time to capture the plasma curves.

The effects of dietary meat consumption on circulating TMAO have also received considerable attention due to the implications for CVD. Meat, notably red meat, is a rich source of L-carnitine, which is metabolized by gut microbiota to TMA. As with egg ingestion, we observed a non-significant trend towards increased postprandial TMAO levels in MetS subjects relative to non-MetS subjects after meat consumption. Most previous studies have shown that the levels of urinary or plasma TMAO and/or TMA are significantly associated with meat intake [31]. In a study by Koeth et al. [14], omnivorous subjects that consumed a 250 g beef steak exhibited a 10-fold rise in plasma TMAO within 4 h, whereas vegans/vegetarians demonstrated a negligible increase, highlighting the critical role of gut microbiota composition in TMAO production. Similar results were also reported from a long-term intervention by Wang et al. (2019) [13]. In that study, subjects consuming red meat (vs. white meat or plant-based protein) in a 4-week randomized trial showed a 2–3-fold increase in fasting plasma TMAO, accompanied by shifts in gut microbial composition favoring TMA synthesis [13]. Conversely, a plant-based diet reduced TMAO levels by suppressing TMA-generating taxa [13].

With the exception of one study [19], little research has focused on analyzing the involvement of the intermediate metabolite TMA in TMAO production. TMA is the direct metabolite generated from the gut-microbiota-mediated metabolism of L-carnitine and choline and hence it can provide insights into the distribution of bacteria in the gut. In this study, subjects with MetS were found to differ from healthy subjects regarding the types of microbes that live in the gastrointestinal tract, which is a characteristic that can profoundly affect metabolism [32] and mediate differences in the TMA formation.

While TMAO is a gut microbiota-dependent metabolite, our findings revealed that subjects with MetS and no evidence of CVD and T2D did not exhibit significant alterations in microbial taxa responsible for TMA production—the critical precursor of TMAO. This absence of microbiota-driven TMA synthesis (e.g., *E. coli*, *Citrobacter*, *Klebsiella pneumoniae*, *Providencia*, *Shigella*, *Achromobacter*, and *Sporosarcina* [9,33]) explains the non-significant postprandial TMAO increase observed in MetS compared to non-MetS subjects, despite their divergent microbial ecology characterized by reduced diversity and pro-inflammatory shifts (e.g., depletion of *Blautia glucerasea* and enrichment of *Ruminococcus torques*). *Ruminococcus torques* is a mucin-degrading bacterium that efficiently degrades human colonic MUC2 [34], a key component of the protective intestinal mucus layer. This activity reduces mucus thickness, increases penetrability, and promotes inflammation-linked pathologies [34,35]. Indeed, *R. torques* is implicated in inflammatory bowel diseases (e.g., Crohn’s disease), in the degradation of the blood group antigen components (A and H) in intestinal glycosphingolipids, and in hemodialysis-related dysbiosis, and is positively associated with TMAO levels [36,37]. Collectively, these results suggest that in early-stage MetS—after excluding confounders such as T2D and CVD which are known to elevate TMAO—the observed gut dysbiosis may be linked to inflammatory pathways independently of TMAO generation. Even considering the limited sample size and the cross-sectional study design, one might assume that the observed TMAO increase is more likely a consequence than a cause of advanced metabolic disorder; a hypothesis that should be confirmed by large longitudinal studies.

A key strength of this study is the combined use of plasma, urine, and fecal samples as this provides a multidimensional perspective on host–microbiota–metabolite interactions. In addition, the focus on individuals with MetS, before the onset of overt CVD or T2D, allowed for the identification of gut microbiota alterations that may occur prior to disease progression. However, the relatively small sample size, particularly in the MetS group, limits the statistical power and generalizability of the results. Furthermore, the cross-sectional study design only captures acute responses and fails to address the long-term dietary effects on TMAO metabolism. Another restriction is the absence of data on the subjects’ background diet and FMO3 genotype which might affect TMAO production. These limitations emphasize the need for larger and well-controlled long-term studies to validate and extend the preliminary findings of current study. Overall, the results should be viewed with a consideration for their various limitations and strengths.

## 5. Conclusions

Our study demonstrates that early MetS, in the absence of advanced comorbidities such as T2D or CVD, does not significantly elevate postprandial TMAO levels following the consumption of choline- or L-carnitine-rich foods (eggs and meatballs). The lack of TMA-generating bacteria in subjects with MetS—despite a distinct dysbiotic profile marked by reduced *Blautia glucerasea* and enriched *Ruminococcus torques*—provides a plausible explanation for the non-significant TMAO elevation. However, this finding should be regarded as exploratory due to the limited sample size. Taken together, these results suggest that in early-stage MetS the observed gut dysbiosis may be linked to inflammatory pathways independently of TMAO generation, meaning that TMAO may represent a consequence rather than an initiator of advanced metabolic dysfunction. This hypothesis should be confirmed by large longitudinal, well-powered studies.

## Figures and Tables

**Table 1:** Kinetic data of plasma methylamines in subjects without (n = 21) and with MetS (n = 12) before and after ingestion of 3 hard-boiled eggs (≈170 g) or 170 g meatballs.

Compound	TMA	TMAO	Carnitine	Choline	Betaine	A. Carnitine
Amount (mg) ^a^ in Ingested Egg				357 ± 16.5	1.7 ± 0.1	-
non-MetS						
C_0_ (µM) ^b^	0.58 ± 0.27	3.32 ± 0.90	30.89 ± 4.87	9.04 ± 1.77	29.12 ± 6.47	6.44 ± 1.84
C_max_ (µM) ^c^	0.61 ± 0.27	3.32 ± 0.90	33.40 ± 4.87	15.28 ± 2.94	45.73 ± 10.21	7.31 ± 2.09
ΔC_max_ (µM) ^d^	0.03 ± 0.38	0.00	2.51 ± 6.89	2.51 ± 6.89	6.24 ± 3.43	0.87 ± 2.79
t_max_ (min) ^e^	120	0	240			
C_360_ ^f^	0.55 ± 0.24	2.87 ± 1.46	30.43 ± 5.35	12.04 ± 2.53	41.02 ± 8.07	7.31 ± 2.09
IAUC^0–360^ (h × µM) ^g^	12.85 ± 15.44	93.39 ± 286.45	652.79 ± 583.61	1447.66 ± 503.44	4045.35 ± 1419.54	167.25 ± 175.02
MetS						
C_0_ (µM) ^b^	0.56 ± 0.22	8.02 ± 12.27	34.76 ± 7.17	9.24 ± 2.21	29.82 ± 9.11	6.32 ± 1.92
C_max_ (µM) ^c^	0.60 ± 0.23	8.02 ± 12.27	37.83 ± 5.40	16.48 ± 4.18	45.68 ± 12.00	7.36 ± 1.55
ΔC_max_ (µM) ^d^	0.04 ± 0.32	0.00	3.07 ± 8.97	7.24 ± 4.73	15.86 ± 15.07	1.04 ± 2.47
t_max_ (min) ^e^	240	0	240	120	240	360
C_360_ ^f^	0.59 ± 0.19	5.69 ± 4.52	36.24 ± 6.03	13.43 ± 2.47	43.35 ± 10.98	7.36 ± 1.55
IAUC^0–360^ (h × µM) ^g^	18.05 ± 19.56	170.34 ± 242.89	889.56 ± 1338.87	1898.78 ± 846.02	3896.75 ± 1928.66	161.06 ± 213.19
Amount (mg) ^a^ in ingested meatballs			54 ± 0.6	66 ± 0.7	16 ± 0.2	
non-MetS						
C_0_ (µM) ^b^	0.60 ± 0.26	5.83 ± 8.72	29.85 ± 6.51	9.14 ± 2.15	29.18 ± 6.56	6.73 ± 2.52
C_max_ (µM) ^c^	0.60 ± 0.26	5.83 ± 8.72	34.30 ± 7.16	10.41 ± 2.58	34.06 ± 7.26	8.22 ± 2.51
Δ C (µM) ^d^	0.00	0.00	4.45 ± 9.68	1.27 ± 3.36	4.88 ± 9.78	1.49 ± 3.56
t_max_ (min) ^e^	0	0	120	120	60	360
C_360_ ^f^	0.52 ± 0.21	3.62 ± 1.81	29.88 ± 5.64	9.03 ± 2.26	27.92 ± 6.25	8.22 ± 2.51
IAUC^0–360^ (h × µM) ^g^	8.24 ± 8.30	56.34 ± 90.44	1047.83 ± 567.72	318.94 ± 241.12	840.67 ± 621.53	147.58 ± 85.30
MetS						
C_0_ (µM) ^b^	0.68 ± 0.38	11.09 ± 16.69	37.49 ± 6.25	9.51 ± 1.98	30.59 ± 8.78	7.21 ± 1.76
C_max_ (µM)^c^	0.68 ± 0.38	11.09 ± 16.69	43.09 ± 7.51	11.60 ± 3.19	35.87 ± 9.93	7.82 ± 2.08
Δ C (µM) ^d^	0.00	0.00	5.60 ± 9.77	2.09 ± 3.75	5.28 ± 13.26	0.61 ± 2.73
t_max_ (min) ^e^	0	0	240	60	60	360
C_360_ ^f^	0.56 ± 0.20	6.15 ± 5.99	38.64 ± 6.70	9.22 ± 1.43	31.58 ± 8.86	7.82 ± 2.08
IAUC^0–360^ (h × µM) ^g^	18.10 ± 26.17	97.76 ± 154.06	1358.18 ± 735.17	555.60 ± 562.90	1331.37 ± 672.95	71.88 ± 68.33

A. Carnitine: Acetyl-L-carnitine. ^a^ Mean content of methylamines (mg) per portion (~170 g) of test food. ^b^ Plasma concentration (µM) before ingestion of test food. ^c^ Maximum plasma concentration. ^d^ Incremental maximum plasma concentration, calculated as C_max_ − C_0_. ^e^ Time (min) of maximum plasma concentration. ^f^ Plasma concentration at 360 min after ingestion of test food (i.e., last sampling). ^g^ Incremental area under the concentration curve (above C_0_) from t = 0 to t = 360 min.

**Table 2:** Urinary excretion of methylamines (µmol/6 h) in subjects without (n = 21) and with MetS (n = 12) after ingestion of 170 g meatballs or 3 hard-boiled eggs (≈170 g).

Variable	Non-MetS (n = 21)	MetS (n = 12)	Mann–Whitney U Test
After egg ingestion			
TMA	2.56 ± 0.97	2.95 ± 1.23	ns
TMAO	109.42 ± 45.86	204.54 ± 214.6	0.05
L-Carnitine	9.10 ± 7.58	11.61 ± 12.07	ns
Choline	20.77 ± 5.80	23.80 ± 5.92	ns
Betaine	33.42 ± 23.80	37.99 ± 8.53	ns
Acetyl-L-carnitine	5.48 ± 4.91	4.50 ± 4.48	ns
After meat ingestion			
TMA	3.42 ± 1.85	4.37 ± 2.99	ns
TMAO	181.00 ± 169.71	222.30 ± 278.49	ns
L-Carnitine	31.07 ± 22.37	44.23 ± 43.02	ns
Choline	14.90 ± 5.96	13.73 ± 6.79	ns
Betaine	27.03 ± 21.96	35.09 ± 19.34	ns
Acetyl-L-carnitine	14.80 ± 11.06	16.59 ± 15.52	ns

Values corrected for urine volume; ns: not significant.

## Data Availability

The original contributions presented in this study are included in the article. Further inquiries can be directed to the corresponding author.

## Abbreviations

The following abbreviations are used in this manuscript:

ATP III	Adult Treatment Panel III
CntA/B	A two-component Rieske-type oxygenase/reductase system converts carnitine to TMA
CutC	Glycyl radical enzyme converts choline to TMA (choline TMA-lyase)
CutD	CutC-activating protein (the activator protein for choline TMA-lyase)
YeaW/X	A two-component Rieske-type oxygenase/reductase system converts betaine to TMA
CVD	Cardiovascular disease
FMO3	Flavin-containing monooxygenase 3
MetS	Metabolic syndrome
MUC2	Mucin 2, oligomeric mucus/gel-forming
NCEP	National Cholesterol Education Program
OPLS-DA	Orthogonal Partial Least Squares Discriminant Analysis
PLS	Partial Least Squares Discriminant Analysis
rho	Spearman correlation coefficients
SCFAs	Short-chain fatty acids
SD	Standard deviation
T2D	Type 2 diabetes
TMAO	Trimethylamine *N*-oxide
TMA	Trimethylamine
IACN	Iodoacetonitrile
ACN	Acetonitrile

## References

[1] Lopez-Candales A., Hernandez Burgos P.M., Hernandez-Suarez D.F., Harris D. (2017). Linking Chronic Inflammation with Cardiovascular Disease: From Normal Aging to the Metabolic Syndrome. J. Nat. Sci..

[2] Cleeman J.I. (2001). Executive Summary of The Third Report of The National Cholesterol Education Program (NCEP) Expert Panel on Detection, Evaluation, and Treatment of High Blood Cholesterol in Adults (Adult Treatment Panel III). JAMA.

[3] Boulangé C.L., Neves A.L., Chilloux J., Nicholson J.K., Dumas M.E. (2016). Impact of the gut microbiota on inflammation, obesity, and metabolic disease. Genome Med..

[4] Pitocco D., Di Leo M., Tartaglione L., De Leva F., Petruzziello C., Saviano A., Pontecorvi A., Ojetti V. (2020). The role of gut microbiota in mediating obesity and diabetes mellitus. Eur. Rev. Med. Pharmacol. Sci..

[5] Le Chatelier E., Nielsen T., Qin J., Prifti E., Hildebrand F., Falony G., Almeida M., Arumugam M., Batto J.M., Kennedy S. (2013). Richness of human gut microbiome correlates with metabolic markers. Nature.

[6] Cani P.D., Everard A., Duparc T. (2013). Gut microbiota, enteroendocrine functions and metabolism. Curr. Opin. Pharmacol..

[7] Tremaroli V., Bäckhed F. (2012). Functional interactions between the gut microbiota and host metabolism. Nature.

[8] El Hage R., Al-Arawe N., Hinterseher I. (2023). The Role of the Gut Microbiome and Trimethylamine Oxide in Atherosclerosis and Age-Related Disease. Int. J. Mol. Sci..

[9] Janeiro M.H., Ramírez M.J., Milagro F.I., Martínez J.A., Solas M. (2018). Implication of Trimethylamine *N*-Oxide (TMAO) in Disease: Potential Biomarker or New Therapeutic Target. Nutrients.

[10] Evans M., Dai L., Avesani C.M., Kublickiene K., Stenvinkel P. (2023). The dietary source of trimethylamine *N*-oxide and clinical outcomes: An unexpected liaison. Clin. Kidney J..

[11] Craciun S., Balskus E.P. (2012). Microbial conversion of choline to trimethylamine requires a glycyl radical enzyme. Proc. Natl. Acad. Sci. USA.

[12] Koeth R.A., Levison B.S., Culley M.K., Buffa J.A., Wang Z., Gregory J.C., Org E., Wu Y., Li L., Smith J.D. (2014). γ-Butyrobetaine Is a Proatherogenic Intermediate in Gut Microbial Metabolism of L-Carnitine to TMAO. Cell Metab..

[13] Wang Z., Bergeron N., Levison B.S., Li X.S., Chiu S., Jia X., Koeth R.A., Li L., Wu Y., Tang W.H.W. (2019). Impact of chronic dietary red meat, white meat, or non-meat protein on trimethylamine *N*-oxide metabolism and renal excretion in healthy men and women. Eur. Heart J..

[14] Koeth R.A., Wang Z., Levison B.S., Buffa J.A., Org E., Sheehy B.T., Britt E.B., Fu X., Wu Y., Li L. (2013). Intestinal microbiota metabolism of l-carnitine, a nutrient in red meat, promotes atherosclerosis. Nat. Med..

[15] Tang W.H.W., Wang Z., Levison B.S., Koeth R.A., Britt E.B., Fu X., Wu Y., Hazen S.L. (2013). Intestinal Microbial Metabolism of Phosphatidylcholine and Cardiovascular Risk. N. Engl. J. Med..

[16] Wang Z., Klipfell E., Bennett B.J., Koeth R., Levison B.S., DuGar B., Feldstein A.E., Britt E.B., Fu X., Chung Y.-M. (2011). Gut flora metabolism of phosphatidylcholine promotes cardiovascular disease. Nature.

[17] Tune J.D., Goodwill A.G., Sassoon D.J., Mather K.J. (2017). Cardiovascular consequences of metabolic syndrome. Transl. Res..

[18] Lent-Schochet D., Silva R., McLaughlin M., Huet B., Jialal I. (2018). Changes to trimethylamine-*N*-oxide and its precursors in nascent metabolic syndrome. Horm. Mol. Biol. Clin. Investig..

[19] Cho C.E., Taesuwan S., Malysheva O.V., Bender E., Tulchinsky N.F., Yan J., Sutter J.L., Caudill M.A. (2017). Trimethylamine-*N*-oxide (TMAO) response to animal source foods varies among healthy young men and is influenced by their gut microbiota composition: A randomized controlled trial. Mol. Nutr. Food Res..

[20] Barrea L., Annunziata G., Muscogiuri G., Di Somma C., Laudisio D., Maisto M., De Alteriis G., Tenore G.C., Colao A., Savastano S. (2018). Trimethylamine-*N*-oxide (TMAO) as Novel Potential Biomarker of Early Predictors of Metabolic Syndrome. Nutrients.

[21] Hefni M.E., Witthöft C.M. (2025). Development and Application of a UPLC–MRM–MS Method for Quantifying Trimethylamine, Trimethylamine-*N*-Oxide, and Related Metabolites in Individuals with and Without Metabolic Syndrome. Separations.

[22] Hefni M.E., Witthöft C.M., Hellström P., Johansson I., Esberg A. (2025). Plasma TMAO Concentrations and Gut Microbiota Composition in Subjects with and Without Metabolic Syndrome: Results from Pilot Study. Metabolites.

[23] Nickols W.A., Kuntz T., Shen J., Maharjan S., Mallick H., Franzosa E.A., Thompson K.N., Nearing J.T., Huttenhower C. (2024). MaAsLin 3: Refining and extending generalized multivariable linear models for meta-omic association discovery. bioRxiv.

[24] RStudioTeam (2020). RStudio: Integrated Development for R.

[25] Ringel C., Dittrich J., Gaudl A., Schellong P., Beuchel C.F., Baber R., Beutner F., Teren A., Engel C., Wirkner K. (2021). Association of plasma trimethylamine *N*-oxide levels with atherosclerotic cardiovascular disease and factors of the metabolic syndrome. Atherosclerosis.

[26] Kuo C.-H., Liu C.-H., Wang J.-H., Hsu B.-G. (2022). Serum Trimethylamine *N*-Oxide Levels Correlate with Metabolic Syndrome in Coronary Artery Disease Patients. Int. J. Environ. Res. Public Health.

[27] Miller C.A., Corbin K.D., da Costa K.A., Zhang S.C., Zhao X.Q., Galanko J.A., Blevins T., Bennett B.J., O’Connor A., Zeisel S.H. (2014). Effect of egg ingestion on trimethylamine-*N*-oxide production in humans: A randomized, controlled, dose-response study. Am. J. Clin. Nutr..

[28] Zhu C., Sawrey-Kubicek L., Bardagjy A.S., Houts H., Tang X., Sacchi R., Randolph J.M., Steinberg F.M., Zivkovic A.M. (2020). Whole egg consumption increases plasma choline and betaine without affecting TMAO levels or gut microbiome in overweight postmenopausal women. Nutr. Res..

[29] DiMarco D.M., Missimer A., Murillo A.G., Lemos B.S., Malysheva O.V., Caudill M.A., Blesso C.N., Fernandez M.L. (2017). Intake of up to 3 Eggs/Day Increases HDL Cholesterol and Plasma Choline While Plasma Trimethylamine-*N*-oxide is Unchanged in a Healthy Population. Lipids.

[30] Zeisel S.H., Wishnok J.S., Blusztajn J.K. (1983). Formation of methylamines from ingested choline and lecithin. J. Pharmacol. Exp. Ther..

[31] Lombardo M., Aulisa G., Marcon D., Rizzo G., Tarsisano M.G., Di Renzo L., Federici M., Caprio M., De Lorenzo A. (2021). Association of Urinary and Plasma Levels of Trimethylamine *N*-Oxide (TMAO) with Foods. Nutrients.

[32] Turnbaugh P.J., Gordon J.I. (2009). The core gut microbiome, energy balance and obesity. J. Physiol..

[33] Rath S., Heidrich B., Pieper D.H., Vital M. (2017). Uncovering the trimethylamine-producing bacteria of the human gut microbiota. Microbiome.

[34] Schaus S.R., Pereira G.V., Luis A.S., Madlambayan E., Terrapon N., Ostrowski M.P., Jin C., Henrissat B., Hansson G.C., Martens E.C. (2024). *Ruminococcus torques* is a keystone degrader of intestinal mucin glycoprotein, releasing oligosaccharides used by *Bacteroides thetaiotaomicron*. mBio.

[35] Desai M.S., Seekatz A.M., Koropatkin N.M., Kamada N., Hickey C.A., Wolter M., Pudlo N.A., Kitamoto S., Terrapon N., Muller A. (2016). A Dietary Fiber-Deprived Gut Microbiota Degrades the Colonic Mucus Barrier and Enhances Pathogen Susceptibility. Cell.

[36] Png C.W., Lindén S.K., Gilshenan K.S., Zoetendal E.G., McSweeney C.S., Sly L.I., McGuckin M.A., Florin T.H.J. (2010). Mucolytic Bacteria with Increased Prevalence in IBD Mucosa AugmentIn VitroUtilization of Mucin by Other Bacteria. Am. J. Gastroenterol..

[37] Shi X., Gao B., Srivastava A., Izzi Z., Abdalla Y., Shen W., Raj D. (2022). Alterations of gut microbial pathways and virulence factors in hemodialysis patients. Front. Cell. Infect. Microbiol..

